# Digital Health Interventions for Diabetes Distress in Type 2 Diabetes: Protocol for a Scoping Literature Review Focused on Equity and Inclusion

**DOI:** 10.2196/85406

**Published:** 2026-04-27

**Authors:** Raul Szekely, Megan Armstrong, Adam W A Geraghty, Judith Edwards, Kate Hardenberg, Joanne Lloyd, Rhiannon Hawkes, Nana Ocran, Mel Ramasawmy, Shoba Poduval, Sophie Turnbull, Charlotte Dack, Jamie Ross

**Affiliations:** 1 Centre for Primary Care, Wolfson Institute of Population Health, Queen Mary University of London, London, United Kingdom; 2 Research Department of Primary Care and Population Health, Institute of Epidemiology and Health Care, University College London, London, United Kingdom; 3 School of Primary Care, Population Sciences and Medical Education, Faculty of Medicine, University of Southampton, Southampton, United Kingdom; 4 Diabetes Psychotherapy Service, Diabetes and Endocrinology, Royal United Hospitals Bath NHS Foundation Trust, Bath, United Kingdom; 5 Public Contributor, Bath, United Kingdom; 6 Centre for Health Psychology, Division of Psychology and Mental Health, University of Manchester, Manchester, United Kingdom; 7 Public Contributor, London, United Kingdom; 8 Centre for Cancer Screening, Prevention, and Early Diagnosis, Wolfson Institute of Population Health, Queen Mary University of London, London, United Kingdom; 9 Centre for Academic Primary Care, Bristol Medical School, University of Bristol, Bristol, United Kingdom; 10 Department of Psychology, University of Bath, Claverton Down, Bath, BA2 7AY, United Kingdom, +44 01225 383843

**Keywords:** digital health interventions, diabetes distress, type 2 diabetes, user experience, engagement, health inequalities, digital exclusion

## Abstract

**Background:**

Managing type 2 diabetes (T2D) requires ongoing attention to diet, physical activity, medication, and blood glucose monitoring. These cumulative demands can lead to diabetes distress, a common emotional strain that affects well-being, self-management, and clinical outcomes. Digital health interventions (DHIs) offer scalable, cost-effective support for both self-management and emotional well-being. However, many DHIs pay limited attention to the needs of underserved groups, who may experience higher distress, face additional barriers to engagement, and are often underrepresented in research. Evidence describing how DHIs are designed, delivered, and experienced across diverse populations remains fragmented, particularly from an equity perspective.

**Objective:**

This mixed methods scoping review aims to map and characterize DHIs addressing the emotional burden of living with T2D and examine how equity and inclusion are considered in these interventions.

**Methods:**

Electronic searches will be conducted in several databases, including MEDLINE, CINAHL, and PsycINFO, as well as gray literature sources. Quantitative, qualitative, and mixed methods studies published since 1998 involving adults with T2D who have used DHIs targeting diabetes distress will be included. Particular attention will be given to equity, including recruitment practices, content adaptations, and provision of digital infrastructure and support. Screening and data extraction will be performed independently by multiple reviewers. Quantitative and qualitative data will be analyzed separately and integrated through descriptive mapping and thematic synthesis to identify trends, gaps, and barriers.

**Results:**

The initial search was completed in August 2025. Comprehensive database and gray literature searches, along with screening, were conducted between September and October 2025. After screening, 68 studies met the inclusion criteria. Data extraction was finalized in January 2026, with the final write-up projected for completion by summer 2026.

**Conclusions:**

This review will identify gaps in existing DHIs and provide guidance to improve their design, inclusivity, and responsiveness to the emotional needs of adults with T2D, particularly those from ethnic minority and other underserved groups, supporting the development of user-centered, equitable solutions.

## Introduction

### Background

Diabetes distress (DD) is a common psychological response to the challenges of self-managing type 2 diabetes (T2D) characterized by feeling overwhelmed, frustrated, guilty, or hopeless [1-4]. DD affects a substantial proportion of adults living with the condition, with prevalence estimates ranging from 36% [1] to nearly 70% over time [5], depending on the measures used and populations studied. These emotional difficulties can undermine a patient’s motivation and confidence to engage in adequate self-management, often worsening glycemic control [6-8], which amplifies the risk of serious diabetes-related complications [9] and places greater burden on health care systems [10-13]. Although it can co-occur with depression, DD is a separate construct and more directly associated with self-management behaviors and glycemic outcomes [6,14-17]. Given its prevalence, impact, and distinct profile, providing effective, tailored support for DD is essential.

Psychological and behavioral interventions have shown promise in reducing DD, and in some cases, improving related outcomes, such as anxiety, depressive symptoms, self-efficacy, and self-management behaviors [18-24]. While much evidence focuses on traditional delivery modes, digital health interventions (DHIs), including apps, websites, telehealth platforms, remote monitoring tools, and wearables, are promoted as scalable and cost-effective solutions that can extend access to care [25]. These interventions are increasingly used to support the self-management and well-being of people with chronic conditions, including T2D [26-37]. In T2D, DHIs have been found to improve glycemic control [36], and in some instances, reduce medication use and the number of outpatient appointments [38]. Emerging evidence also suggests that artificial intelligence–enabled conversational agents or chatbots may facilitate diabetes self-management by providing personalized guidance, psychoeducation, and emotional support [39-42], although research in this area remains at an early stage.

Despite growing interest in digital approaches, only a handful of systematic reviews have explored digital or technology-based interventions targeting DD or similar outcomes [31-33,43]. These reviews suggest that DHIs may produce modest improvements in DD, but the evidence base remains heterogeneous, with considerable variations in intervention types, delivery formats, and outcome measures. Reviews have also highlighted inconsistent reporting of intervention components and limited use of theoretical frameworks, making it difficult to determine why some interventions appear more effective than others.

Behavior change techniques (BCTs) are considered the active components of behavioral interventions, representing the replicable elements that alter causal processes underlying behavior change (eg, beliefs, self-regulation, and motivation) [44,45]. As such, they are recognized as key mechanisms through which interventions, including DHIs, achieve effects on behavior and health outcomes [46-48]. In addition to BCTs, DHI may incorporate therapeutic components, such as cognitive-behavioral strategies, mindfulness, or psychoeducation [32,49-51]. However, existing studies often provide limited detail on how these elements are integrated within digital interventions targeting DD or how combinations of techniques may influence engagement and outcomes [31].

Important questions also remain regarding the accessibility and real-world relevance of DHIs, particularly for underserved populations who may face barriers to digital participation, including people facing socioeconomic disadvantage, ethnic and racial minority groups, individuals living in rural or remote areas, people with disabilities, and those with limited digital literacy or access to technology [52]. These groups may bear a disproportionate burden of DD [53-58]; yet, they are often underrepresented in digital health research and intervention development [59-61]. If underserved groups are not adequately considered during design, evaluation, and implementation, DHIs may fail to address their needs or risk exacerbating existing health inequalities.

Qualitative research has begun to explore how people with T2D perceive and engage with relevant digital tools and interventions, including barriers and facilitators to access and use [27,55,56,62-65]. For example, Turnbull et al [56] have shown that factors, such as limited digital skills, financial costs, and lack of access to technology, can create barriers to using digital health tools for diabetes self-management, particularly among socially disadvantaged groups. Other work has highlighted that individuals experiencing DD may feel digitally alienated or prefer human interaction over technology-based support, which can influence engagement with DHIs [65]. However, these insights have not yet been synthesized alongside evidence on intervention design, therapeutic components, and outcomes.

Given the diversity and heterogeneity of existing evidence, a mixed methods scoping review is needed to provide a broad, exploratory mapping of DHIs for DD (eg, the different types of interventions, their content, and how they are designed, structured, and delivered), identify their active components, including therapeutic strategies and BCTs (eg, the specific mechanisms or strategies embedded in interventions that are theorized to produce changes in self-management, emotional well-being, or related behaviors), and examine how diverse populations experience and engage with these interventions (eg, how users interact with, adhere to, perceive, and respond to interventions, including factors that support or hinder engagement). Particular attention will be paid to how issues of equity and inclusion are addressed across the literature, both in research practices and in the design and delivery of DHIs.

A preliminary search carried out on February 7, 2025 identified several relevant systematic reviews [18,19,31,33], but no reviews synthesizing qualitative and mixed methods evidence relating to intervention design and delivery, therapeutic content and behavior change strategies, user experience and engagement, or equity-related considerations in DHIs for DD, confirming the novelty and appropriateness of this work.

### Aim and Objectives

This review aims to map and characterize DHIs targeting DD in adults with T2D, with special attention to how equity and inclusion are considered.

The specific objectives of the review are as follows:

- To describe the landscape of DHIs for DD in adults with T2D in terms of their design, structure, content, and delivery, capturing how interventions are developed and implemented to support emotional well-being and self-management;
- To identify active components within these DHIs, including therapeutic strategies and BCTs, and explain how these components are intended to influence DD and related cognitive, affective, or behavioral outcomes;
- To explore user experience and engagement, including how people with T2D interact with, adhere to, perceive, and respond to these interventions, as well as factors that facilitate or hinder use;
- To assess equity and inclusion, including reporting of participant demographics, targeted recruitment and inclusion/exclusion practices, access to digital technologies, subgroup analyses of differential effects, and whether interventions are culturally or linguistically adapted to improve accessibility, relevance, and responsiveness for underserved populations;
- To map outcomes, summarizing how DD and related psychological or behavioral outcomes are measured, and describe the reported impact of DHIs on these outcomes; and
- To identify trends, gaps, and opportunities across literature, highlighting areas where evidence is limited, inconsistent, or missing, to inform the design, delivery, evaluation, and reporting of future research and practice efforts.

## Methods

A mixed methods scoping review will be conducted in line with the Joanna Briggs Institute (JBI) guidance [66] and reported according to the PRISMA-ScR (Preferred Reporting Items for Systematic Reviews and Meta-Analyses extension for Scoping Reviews) guidelines (Checklist 1) [67].

### Population-Concept-Context

This review and its inclusion criteria will be guided by the population-concept-context framework [68].

#### Population

This review will include studies involving adults diagnosed with T2D.

#### Concept

The review will examine DHIs, such as mobile apps, websites, adaptive/interactive messaging systems, telehealth platforms, remote monitoring tools, wearable devices, virtual reality, and online programs, designed to prevent or reduce diabetes-related distress in people with T2D.

#### Context

The review focuses on DHIs for DD in adults with T2D, whether linked to health care or community contexts or accessed entirely independently, including in research trials without health care professional or community involvement, as long as they are relevant to the needs of the target population. Interventions may be self-guided, peer-supported, or prescribed/supported/facilitated by health care staff or other relevant professionals.

### Types of Sources

This review will include primary research studies with qualitative, quantitative, or mixed methods designs. Eligible sources include randomized controlled trials, quasi-experimental studies, cohort studies, cross-sectional studies, qualitative research, and mixed methods evaluations.

Gray literature sources, such as reports from public health organizations or nongovernmental organizations, doctoral theses and dissertations, implementation case studies, and conference abstracts (where sufficient data are reported), will also be included.

Systematic reviews, scoping reviews, protocols, editorials, opinion pieces, and purely theoretical papers will be excluded.

No restrictions will be placed on publication language at the point of search. Studies published from 1998 onward will be included to reflect the emergence of digital health technologies.

### Eligibility Criteria

Table 1 presents the criteria that will be used during the screening phases to determine the potential eligibility of studies.

**Table 1.** Eligibility criteria for study screening.

Criterion	Description
Population	Studies involving adults aged 18 years or older who have been diagnosed with T2D^a^, including those with comorbid conditions, will be included. Studies focusing exclusively on type 1 diabetes, gestational diabetes, or other forms of diabetes will be excluded, owing to differing psychological and behavioral management pathways. Studies that report subgroup analyses or stratified data for adults with T2D, even if other diabetes types are included in the wider sample, will also be included.
Intervention	Interventions must target the prevention or reduction of diabetes distress, either as a primary or ancillary aim, or explicitly include content related to the emotional management of T2D. Interventions may encompass mobile apps, websites, adaptive or interactive digital messaging systems, telehealth platforms with digital features, remote monitoring tools, wearable devices, virtual reality, online programs, AI^b^, and similar digital tools/technologies. Interventions delivered solely by telephone, face-to-face, paper-based materials, or via remote channels where the only digital aspect is the mode of delivery (eg, Zoom and WhatsApp) will be excluded, unless the intervention also includes a substantive digital component (eg, app features, interactive content, structured online modules, etc). Interventions can be self-guided, peer-supported, or prescribed/supported/facilitated by health care or other relevant professionals (eg, GPs^c^, diabetes specialists, nurses, educators, mental health, allied health professionals, coaches, researchers, etc).
Outcomes	Diabetes distress—studies are included if they assess the impact of the DHI^d^ on the emotional or psychological burden of living with T2D. *Quantitative studies*: must use validated diabetes distress scales (eg, PAID^e^ and DDS)^f^ *Qualitative studies*: must explore how the intervention affects the emotional management of T2D User experience and/or engagement—studies are included only if the intervention content explicitly addresses emotional or psychological aspects of people living with T2D, even if diabetes distress is not measured. *User experience:* users’ perceptions and responses that result from actual or anticipated use of the DHI, which may include acceptability, satisfaction, credibility, usability, or perceived impact [69,70] *Engagement:* the extent and pattern of users’ interaction with the DHI, which may be assessed using objective metrics (eg, frequency, duration, and feature use) or self-reported behavioral involvement [71,72]
Setting	Interventions must be relevant to contexts in which adults with T2D may experience diabetes distress, including health care settings (eg, primary care, specialist clinics, hospital inpatient or outpatient services, mental health services, etc), community-based settings (eg, local health programs, charity-run initiatives, etc), and other settings (eg, at-home, online environments, etc). Both research-based interventions and those implemented in real-world practice will be included, provided they address the needs of adults with T2D experiencing diabetes distress. Studies from all income settings will be included.
Study design	Primary research using qualitative, quantitative, or mixed methods approaches will be included. Reviews, protocols, editorials, opinion pieces, and theoretical papers will be excluded.
Publication characteristics	Studies published from 1998 onward will be included. Gray literature sources will be included. All languages considered.

a T2D: type 2 diabetes.

b AI: artificial intelligence.

c GPs: general practitioners.

d DHI: digital health intervention.

e PAID: Problem Areas in Diabetes Scale.

f DDS: Diabetes Distress Scale.

### Search Strategy

#### Three-Step Search Strategy

This scoping review follows the 3-step search strategy recommended by the JBI to ensure comprehensiveness and rigor [66]. Each phase of the strategy is designed to iteratively build and refine the evidence base, beginning with exploratory keyword discovery and culminating in exhaustive database and gray literature searching.

The search strategy was developed by the first author (RS) with guidance from the senior author (JR) and informed by the PRESS (Peer Review of Electronic Search Strategies) guidelines [73], which were consulted to support clarity, completeness, and precision in the approach. Following this, the strategy was reviewed by a health science librarian and discussed with the wider research team before finalization.

#### Step 1: Initial Limited Search

An initial exploratory search was conducted in 2 databases, namely MEDLINE (Ovid) and CINAHL (EBSCO), using terms related to T2D (“type 2 diabetes” or “T2D” or “adults with diabetes”), DD (“diabetes distress” or “emotional burden” or “psychosocial distress”), and DHI (“digital health” or “mobile app” or “mHealth” or “wearables” or “telehealth” or “eHealth” or “web-based intervention” or “remote monitoring” or “virtual reality”).

The titles, abstracts, and indexing of retrieved records were reviewed to generate a list of key search terms and variations. These included both controlled vocabulary and free-text terms, capturing terminological variations across the literature. In addition, the search strategies from literature reviews on related topics were consulted to ensure comprehensive term capture.

#### Step 2: Comprehensive Database Search

Using the terms and insights from Step 1, a comprehensive and tailored search strategy was developed for the following databases: MEDLINE (Ovid), Embase (Elsevier), CINAHL (EBSCO), PsycINFO (EBSCO), Cochrane Library, Epistemonikos, Europe PMC, TRIP Pro, and OTseeker.

Boolean operators (AND/OR), truncation (eg, diabet*), and proximity operators will be used to maximize sensitivity and specificity. Search strings will be adapted to the indexing systems and syntax of each database. Searches will, where applicable, target relevant fields, including titles, abstracts, keywords, and controlled vocabulary. The full search strategy for MEDLINE (Ovid) is displayed in Table 2, while the adapted search strategies for the remaining databases are provided in Multimedia Appendix 1.

**Table 2.** MEDLINE (Ovid) search strategy.

Line number	Search string	Search fields	Hits
1	Diabetes Mellitus, Type 2.sh.	MeSH	191,716
2	(“T2DM” or “T2D” or “type 2 diabetes” or “type two diabetes” or (non-insulin-dependent adj2 diabetes) or “NIDDM” or (“adult-onset” adj2 diabetes)).af.	Title/abstract	208,426
3	1 or 2	Combined	263,462
4	(Psychological Distress or Psychological Well-Being).sh.	MeSH	7052
5	(diabetes adj2 (distress or stress or self-care or self-efficacy or empowerment or emotion* or “quality of life” or well-being or wellbeing or anxiety or depression)).ti,ab.	Title/abstract	9226
6	((psychological or psychosocial or emotional or psychophysical) adj2 (distress or stress or burden or well-being or wellbeing or health or outcomes)).ti,ab.	Title/abstract	132,771
7	4 or 5 or 6	Combined	142,331
8	(Telemedicine or Digital Health or Virtual Reality or Internet-Based Intervention or Mobile Applications or Technology).sh.	MeSH	87,213
9	blog*.ti,ab.	Title/abstract	2721
10	(computer adj2 (app* or therap* or intervention* or program* or communication*)).ti,ab.	Title/abstract	24,829
11	cybertherapy.ti,ab.	Title/abstract	26
12	(digital adj2 (app* or health or intervention* or service* or solution* or tool* or program* or technolog*)).ti,ab.	Title/abstract	31,843
13	(eHealth or e-Health).ti,ab.	Title/abstract	8658
14	e-Mental Health.ti,ab.	Title/abstract	361
15	(e-counsel* or ecounsel*).ti,ab.	Title/abstract	36
16	(e-psychotherap* or epsychotherap*).ti,ab.	Title/abstract	3
17	“email therapy.”ti,ab.	Title/abstract	3
18	forum*.ti,ab.	Title/abstract	20,829
19	(internet adj2 intervention*).ti,ab.	Title/abstract	1960
20	mHealth.ti,ab.	Title/abstract	7381
21	(mobile adj2 (app* or health or support or technolog* or program* or intervention* or tool*)).ti,ab.	Title/abstract	27,393
22	(online adj2 (intervention* or therap* or system* or service* or support or community or tool* or program*)).ti,ab.	Title/abstract	22,388
23	(portable adj2 (app* or technolog*)).ti,ab.	Title/abstract	1657
24	(remote adj2 (coach* or counsel* or psych* or support or intervention* or monitor*)).ti,ab.	Title/abstract	9102
25	SMS.ti,ab.	Title/abstract	9381
26	messag*.ti,ab.	Title/abstract	92,324
27	telecare.ti,ab.	Title/abstract	866
28	teleconsultation.ti,ab.	Title/abstract	1830
29	telehealth.ti,ab.	Title/abstract	15,186
30	teleintervention.ti,ab.	Title/abstract	23
31	telemedicine.ti,ab.	Title/abstract	22,766
32	telemonitoring.ti,ab.	Title/abstract	2613
33	telepsych*.ti,ab.	Title/abstract	1204
34	telerehabilitation.ti,ab.	Title/abstract	2410
35	telecommunication.ti,ab.	Title/abstract	3480
36	((telephone or phone) adj2 (app* or intervention* or therap* or system* or service* or support or tool* or program*)).ti,ab.	Title/abstract	9572
37	(technolog* adj2 (intervention* or therap* or support or tool* or program*)).ti,ab.	Title/abstract	17,511
38	website*.ti,ab.	Title/abstract	47,719
39	(web adj2 (intervention* or program* or tool*)).ti,ab.	Title/abstract	8966
40	or/8‐39	Combined	386,962
41	3 and 7 and 40	Combined	388
42	limit 41 to yr=“1998‐2025”	Limit	386

The search will remain iterative, with refinements made as familiarity with the literature evolves. Any modifications to search terms or strategies during the process will be tracked and documented for transparency and to support reproducibility of the review.

#### Step 3: Gray Literature and Reference Searching

To ensure that important nonindexed sources are not missed, targeted searches for gray literature will be conducted. This includes dedicated databases, such as the Conference Proceedings Citation Index, Google Scholar (first 200 results), and the World Health Organization (WHO) Global Index Medicus; doctoral theses and dissertations (eg, ProQuest Dissertations and Theses Global); organizational and government reports (eg, WHO and National Institute for Health and Care Excellence); clinical trial registries (eg, The International Clinical Trials Registry Platform, ClinicalTrials.gov, and International Standard Randomised Controlled Trial Number Registry); repositories and preprint servers (eg, ScienceOpen and Open Science Framework Preprints); and websites of diabetes organizations (eg, Diabetes UK, The European Association for the Study of Diabetes, World Diabetes Foundation, and International Diabetes Foundation). The same eligibility criteria that apply to peer-reviewed sources will also apply to gray literature. Only sources reporting empirical findings, evaluations, or implementation insights using qualitative, quantitative, or mixed methods approaches will be included. Commentary-only documents, such as opinion pieces, editorials, protocols, reviews, theoretical papers, or policy documents without empirical data, will be excluded.

Furthermore, backward and forward citation chaining will be conducted using Google Scholar and/or Web of Science, and reference lists of all included studies will be manually screened to identify any other potentially relevant records. Where necessary, corresponding authors will be contacted to request additional data and/or materials.

### Source of Evidence Selection

Following the completion of the multiphase search strategy outlined previously, all retrieved search results will be imported into Covidence (a Cochrane technology platform designed and recommended for management of reviews) for deduplication and screening. Screening will be performed in two sequential stages: (1) title and abstract screening and (2) full-text screening.

Prior to formal screening, a sample of 25 titles and abstracts will be selected for pilot testing. Two reviewers will independently screen this sample using the predefined eligibility criteria and a shared definitions guide. The team will meet to check discrepancies and clarify any ambiguous inclusion criteria. Refinements to the screening guide will be made if needed.

Screening will commence only after a minimum absolute inter-rater agreement of 75% is achieved across the team, in order to ensure consistency in decision-making and interpretation of eligibility criteria.

All remaining titles and abstracts will be screened independently by at least 2 reviewers. Records will be assessed for their potential relevance based on the inclusion criteria, and irrelevant studies will be excluded at this stage. Reviewers will be blinded to each other’s decisions to minimize bias. Discrepancies will be resolved through consensus or by involving a third reviewer.

Full-text articles of all studies deemed potentially eligible will be retrieved and reviewed in detail by a minimum of 2 reviewers. Each study will be evaluated independently against all inclusion criteria. As before, discrepancies will be resolved through discussion or by involving a third reviewer.

An audit trail of the selection process will be maintained throughout, and the final report will include a PRISMA flow diagram. Separate appendices will include sources with full bibliographic details and excluded full-text sources with justifications for exclusion.

### Data Extraction

Data extraction will be guided by a standardized form, which will be piloted on 5 studies by the review team to check inter-rater reliability, relevance, and completeness of the fields. Following this pilot, the project’s patient and public involvement (PPI) panel, consisting of a diverse group of people with lived experiences of T2D, will be consulted to provide feedback on the form, including suggestions for additional fields or refinements that better reflect the experiences and priorities of adults with T2D. Modifications will be made based on both pilot testing and PPI input to ensure clarity, efficiency, and relevance before proceeding to full extraction. Modifications will be made based on both pilot testing and PPI input to ensure clarity, efficiency, and relevance before proceeding to full extraction.

The form will capture bibliographical and methodological detail (eg, author, publication year, title, source of publication, country, study design, recruitment methods, and setting), participant demographics (eg, age, gender, ethnicity, language, socioeconomic status, education level, duration of diabetes, treatment type, and proxies for digital exclusion), DHI features (eg, name and version, focus, format, content, delivery mode, timing, duration, frequency, accessibility, etc), therapeutic strategies and BCTs, DD measures and findings, and user experience and engagement data.

Data extraction will then be completed by at least 1 reviewer, with 1 or more reviewers independently verifying the accuracy of the data extraction for a random sample of a minimum of 25% of included studies. Discrepancies will be resolved through discussion, or if necessary, by consulting a third reviewer.

### Data Synthesis

Data from all included studies will be synthesized descriptively to provide a comprehensive overview of DHIs for DD in adults with T2D.

#### Bibliographic and Methodological Details

Frequencies, proportions, ranges, and means will be calculated at the study level to summarize bibliographic trends. Structured tables will present publication characteristics, study design, recruitment strategies, and eligibility criteria, providing a descriptive overview of the literature. Narrative synthesis will describe patterns in publication, study designs, recruitment methods, and reporting gaps, as well as heterogeneity across studies. Charts may be used selectively in future reporting to visualize trends in publication year, geographic distribution, or study design.

#### Participant Characteristics

Participant sociodemographic and clinical characteristics will be synthesized at the study level. Continuous variables (eg, age and diabetes duration) will be summarized using means, SDs, medians, and ranges. Categorical variables (eg, sex/gender, ethnicity, education, and employment) will be summarized using frequencies and proportions. Equity-relevant characteristics will be considered through the lens of the PROGRESS-Plus (Place, Race, Occupation, Gender, Religion, Education, Socioeconomic Status, Social Capital, and Plus) framework [74-76], which provides a structured way to examine factors influencing health inequalities. Extracted participant demographics (eg, age, sex/gender, ethnicity, socioeconomic status, language, religion, and disability) will be mapped to PROGRESS-Plus categories to identify patterns of inclusion and gaps in representation across studies. Narrative synthesis will highlight heterogeneity in participant profiles, trends in inclusion of underserved groups, and implications for engagement, accessibility, and intervention reach. Visualizations, such as bar charts or histograms, may illustrate distributions or highlight reporting gaps.

#### DHI Characteristics

DHI characteristics will be synthesized at the intervention level, summarizing focus, content, delivery mode, duration, onboarding processes, human or peer support, and equity-focused adaptations. Narrative synthesis will be used to describe patterns, common features, and variations across interventions, supported by structured tables presenting these characteristics.

#### Therapeutic Content and BCTs

BCTs are defined as the smallest components of an intervention that are observable, replicable, and capable of changing behavior [44,45]. In this review, BCTs will be coded at the intervention level using the BCT ontology [77], which builds on the earlier BCT taxonomy v1 [44] by providing a more comprehensive, structured, and precise classification. The ontology contains 281 BCTs organized hierarchically into 20 higher-level groups across 5 levels. Level 1 categories are the broadest groupings of BCTs, capturing major types of intervention strategies, such as goal-directed strategies, monitoring, social support, behavioral experiments, etc. For each intervention, the presence or absence of these level 1 categories will be recorded, and frequencies across interventions will be summarized in a table to show which techniques are most commonly used.

Therapeutic strategies (eg, cognitive-behavioral therapy, motivational interviewing, mindfulness, and problem-solving therapy) will also be summarized at the intervention level. Frequencies of use across interventions will be reported, and a narrative description will illustrate how each strategy was operationalized, including the types of activities, exercises, or content delivered to participants.

#### DD Measures and Outcomes

Quantitative data on DD will be extracted and synthesized at the study level, including measurement instruments, baseline and follow-up values, and reported statistical analyses. Studies will be classified according to whether DD was a primary or secondary outcome. Both controlled and uncontrolled studies will be described, noting within-group and between-group changes, effect sizes where reported, and any relevant subgroup analyses (eg, higher baseline distress and intervention intensity). Narrative synthesis will summarize patterns of effect, with commentary on intervention components or participant factors associated with greater reductions in DD.

Qualitative data on DD will also be captured, recording participants’ experiences of distress, coping strategies, and the impact of interventions. Thematic narrative synthesis will summarize key findings, including emotional responses following diagnosis, benefits of structured guidance and human support, and potential mechanisms through which DHIs alleviate distress.

#### User Experience and Engagement

User experience and engagement will be synthesized across studies and interventions. Patterns of usability, acceptability, adherence, and engagement will be described in relation to intervention features (eg, content complexity and human support), participant characteristics, and equity considerations (eg, digital literacy and accessibility). Evidence from self-report measures, qualitative interviews, and system-generated metrics (eg, logins, module completion, diary entries, and interactions with coaches) will be integrated narratively, focusing on factors that support or hinder engagement and sustained use.

### Data Integration

To address the overarching research questions, quantitative, qualitative, and intervention-level findings will be integrated using a convergent approach suitable for mixed methods scoping reviews. For each included study and intervention, a standardized summary sheet will be created containing key quantitative findings, including changes in DD outcomes and engagement metrics, qualitative findings, such as participants’ experiences, perceived barriers, and facilitators, and intervention-level characteristics encompassing focus, content, delivery mode, duration, human or peer support, and equity-focused adaptations. These summary sheets will also capture the presence and frequency of BCTs according to level 1 categories of the BCT ontology and the use of specific therapeutic strategies (eg, cognitive-behavioral therapy, motivational interviewing, mindfulness, and problem-solving therapy), alongside any equity-relevant participant characteristics identified through the PROGRESS-Plus framework.

The summary sheets will be compared side by side and integrated into a master matrix, with rows representing studies or interventions and columns representing quantitative outcomes, qualitative themes, BCT and therapeutic content, participant characteristics, and user engagement patterns. NVivo (Lumivero) will facilitate the coding of qualitative data and enable cross-domain queries, while Excel will be used to summarize frequencies, trends, and patterns across studies. Integration will focus on identifying convergence, divergence, and gaps across different data types. Convergence will be defined as multiple strands supporting similar patterns or findings, divergence as conflicting or inconsistent evidence, and gaps as areas where evidence is limited or absent.

Following integration, a conceptual framework will be developed to depict potential links between intervention design features, equity dimensions, user experience and engagement, and patterns of DD outcomes. This framework will serve as a tool to understand how different digital health interventions may work for diverse populations in varying contexts. The integrated findings and conceptual framework will then inform the derivation of equity-sensitive guiding principles, which will be used to generate practical recommendations for the design, delivery, and evaluation of future interventions for DD.

Finally, the review will identify evidence gaps across quantitative, qualitative, and intervention-level domains, including underreported outcomes, limited equity analysis, or incomplete reporting of engagement or intervention features. These identified gaps will highlight priorities for future research and guide the refinement of intervention development.

### Quality Appraisal

As this is a scoping review, a formal quality appraisal is not standard practice and will not be undertaken.

### Ethical Considerations

This review does not require ethical approval as it involves the secondary analysis of publicly available data.

## Results

The protocol was developed between June and August 2025. The initial search was completed in August 2025, followed by the comprehensive database search in the same month. Eligibility criteria were piloted independently by 2 reviewers (RS and MR) in September 2025 on a sample of 25 titles and abstracts, yielding an inter-rater agreement of 80%. Discrepancies were resolved by consensus, and criteria were refined for clarity. The gray literature search was conducted in September 2025, and screening of titles/abstracts and full texts was carried out by a team of reviewers (RS, JE, MR, and SP) from September to October 2025.

The data extraction form was created and refined with input from PPI members at the end of November 2025. A pilot of data extraction was undertaken at the beginning of December 2025, followed by backward and forward citation searching. Data extraction was completed by January 2026, while data synthesis and integration are expected to be finalized by April 2026. The final manuscript write-up is projected to be completed by June 2026.

The searches identified 4854 references from databases and registers and 16 additional references from other sources. After removal of 1278 duplicates, 3592 records were screened at the title and abstract level. Of these, 374 full texts were sought for retrieval. Nine of these could not be obtained, leaving 365 studies that were assessed for eligibility. From these, 68 studies met the eligibility criteria and were included in the review.

## Discussion

### Anticipated Contributions and Implications

There is growing evidence that DHIs can help support emotional well-being and reduce DD [32,37,43], which is a known psychosocial risk factor for poor self-management and adverse health outcomes among people living with T2D [2,6]. Their flexibility and scalability make them particularly well suited for use in health care settings, such as primary care, community clinics, and outpatient services, where resources are often stretched and patient needs varied.

This review focuses on both *who* current DHIs for DD reach and *how* they are designed, in order to assess whether they address the needs of underserved populations, such as ethnic minority communities, socioeconomically disadvantaged groups, and others at risk of digital exclusion. In doing so, the review will identify DHI features, user experiences, and engagement patterns that could inform the design and delivery of more equitable interventions. For example, the review will examine whether people with lived experience were involved in the development process; whether content was adapted for cultural or linguistic relevance; whether accessibility features were incorporated to accommodate physical, sensory, or neurodiverse needs; whether digital devices, internet access, or technical support were provided; and whether tailored outreach or engagement strategies were employed to engage diverse populations.

The findings will also highlight trends and gaps in equity-related research practices. This includes examining participant recruitment strategies and whether eligibility criteria unintentionally exclude specific groups. The review will also assess the reporting of demographic characteristics, such as age, ethnicity, socioeconomic status, and other equity-relevant variables, and evaluate whether subgroup analyses were conducted to explore differences in outcomes across populations. These insights will provide guidance for more inclusive research efforts and help ensure that underserved populations are adequately represented in future studies.

The findings of this review, together with the findings of a qualitative study conducted in parallel, are expected to contribute to a set of practical, equity-sensitive design and delivery principles to guide the codevelopment of a digital intervention for managing DD in T2D, targeted at underserved communities in England [78]. Moreover, this intervention is being developed specifically for delivery in NHS primary care, the first point of contact in the UK health care system encompassing general practices and other community-based services, and aims to improve access to psychosocial support while alleviating pressure on frontline services.

### Dissemination Strategy

The findings from the scoping review will be disseminated through multiple channels to maximize their relevance, reach, and impact, including peer-reviewed journal publications, conference presentations (eg, in digital health, primary care, and implementation science), and briefings and workshops for digital health developers, NHS stakeholders, and community organizations.

In addition, plain language summaries and visual infographics will be developed in partnership with the PPI panel members to make sure that the findings are accessible and relevant to service users, patient groups, and community stakeholders. This will help ensure that the voices and needs of people with T2D, especially those from underserved communities, remain central to the design and delivery of future interventions.

### Strengths and Limitations

This protocol outlines a mixed methods scoping review led by a multidisciplinary team of academic, clinical, and lived experience experts, which ensures that the review is informed by the real-world needs of people with T2D and remains relevant to both research and practice. Furthermore, the review proposed here adopts an equity-focused stance, aiming to understand whether, and how, the needs of underserved communities, including those at risk of digital exclusion, have been considered in the design and delivery of DHIs for DD. In parallel, the review seeks to examine equity-related research practices across the available literature to determine whether studies have adequately included underserved populations and reported relevant outcomes. In addition, by drawing on a wide range of study designs and sources, and including research from varied settings and populations, the review is set to capture a more complete picture of DHI-related design features, user experience, and engagement, and factors across diverse contexts. However, while patterns and trends that appear to support engagement or positive user experience may be identified, the nature of this review does not allow firm or causal conclusions about what works or the factors driving DHIs’ effectiveness in improving DD. Anticipated heterogeneity in intervention types and outcomes may further limit direct comparability between studies. In addition, because study quality will not be formally assessed, the review is limited to mapping and describing the available evidence rather than evaluating the robustness of the findings.

### Conclusion

Digital interventions aimed at alleviating DD are gaining attention as promising approaches to support the emotional well-being of people with T2D. However, little is known about whether and how they address the needs of groups who could benefit most but are paradoxically at greatest risk of exclusion. This scoping literature review will map the existing evidence on the design and content of DHIs, including active components, user experience and engagement, and impact on DD, as well as how equity considerations are reported across studies. The findings will help guide the development of more inclusive, user-centered, and equitable interventions and promote better research practices to ensure that underserved populations are adequately represented, supporting both accessibility and meaningful engagement.

## Supplementary material

10.2196/85406 Multimedia Appendix 1 Comprehensive search strategy.

10.2196/85406 Checklist 1 PRISMA-ScR (Preferred Reporting Items for Systematic reviews and Meta-Analyses extension for Scoping Reviews) checklist.

## Abbreviations

BCT: behavior change technique
DHI: digital health intervention
JBI: Joanna Briggs Institute
NHS: National Health Service
PPI: patient and public involvement
PRESS: Peer Review of Electronic Search Strategies
PRISMA-ScR: Preferred Reporting Items for Systematic Reviews and Meta-Analyses extension for Scoping Reviews
PROGRESS-Plus: Place, Race, Occupation, Gender, Religion, Education, Socioeconomic Status, Social Capital, and Plus
T2D: type 2 diabetes
WHO: World Health Organization
