# Supplementary material for: Digital Health Interventions for Diabetes Distress in Type 2 Diabetes: Protocol for a Scoping Literature Review Focused on Equity and Inclusion
Source: JMIR Res Protoc. 2026 Apr 27;15:e85406. doi: 10.2196/85406 (PMC13120537; doi:10.2196/85406)
Supplement: Multimedia Appendix 1 [file resprot-v15-e85406-s001.docx]

**MEDLINE (Ovid) search strategy**

| **Line no** | **Search string** | **Search fields** | **Hits** |
| --- | --- | --- | --- |
| #1 | Diabetes Mellitus, Type 2.sh. | MeSH | 191,716 |
| #2 | ("T2DM" or "T2D" or "type 2 diabetes" or "type two diabetes" or (non-insulin-dependent adj2 diabetes) or "NIDDM" or ("adult-onset" adj2 diabetes)).af. | Title/abstract | 208,426 |
| #3 | 1 or 2 | Combined | 263,462 |
| #4 | (Psychological Distress or Psychological Well-Being).sh. | MeSH | 7,052 |
| #5 | (diabetes adj2 (distress or stress or self-care or self-efficacy or empowerment or emotion* or "quality of life" or well-being or wellbeing or anxiety or depression)).ti,ab. | Title/abstract | 9,226 |
| #6 | ((psychological or psychosocial or emotional or psychophysical) adj2 (distress or stress or burden or well-being or wellbeing or health or outcomes)).ti,ab. | Title/abstract | 132,771 |
| #7 | 4 or 5 or 6 | Combined | 142,331 |
| #8 | (Telemedicine or Digital Health or Virtual Reality or Internet-Based Intervention or Mobile Applications or Technology).sh. | MeSH | 87,213 |
| #9 | blog*.ti,ab. | Title/abstract | 2,721 |
| #10 | (computer adj2 (app* or therap* or intervention* or program* or communication*)).ti,ab. | Title/abstract | 24,829 |
| #11 | cybertherapy.ti,ab. | Title/abstract | 26 |
| #12 | (digital adj2 (app* or health or intervention* or service* or solution* or tool* or program* or technolog*)).ti,ab. | Title/abstract | 31,843 |
| #13 | (eHealth or e-Health).ti,ab. | Title/abstract | 8,658 |
| #14 | e-Mental Health.ti,ab. | Title/abstract | 361 |
| #15 | (e-counsel* or ecounsel*).ti,ab. | Title/abstract | 36 |
| #16 | (e-psychotherap* or epsychotherap*).ti,ab. | Title/abstract | 3 |
| #17 | "email therapy".ti,ab. | Title/abstract | 3 |
| #18 | forum*.ti,ab. | Title/abstract | 20,829 |
| #19 | (internet adj2 intervention*).ti,ab. | Title/abstract | 1,960 |
| #20 | mHealth.ti,ab. | Title/abstract | 7,381 |
| #21 | (mobile adj2 (app* or health or support or technolog* or program* or intervention* or tool*)).ti,ab. | Title/abstract | 27,393 |
| #22 | (online adj2 (intervention* or therap* or system* or service* or support or community or tool* or program*)).ti,ab. | Title/abstract | 22,388 |
| #23 | (portable adj2 (app* or technolog*)).ti,ab. | Title/abstract | 1,657 |
| #24 | (remote adj2 (coach* or counsel* or psych* or support or intervention* or monitor*)).ti,ab. | Title/abstract | 9,102 |
| #25 | SMS.ti,ab. | Title/abstract | 9,381 |
| #26 | messag*.ti,ab. | Title/abstract | 92,324 |
| #27 | telecare.ti,ab. | Title/abstract | 866 |
| #28 | teleconsultation.ti,ab. | Title/abstract | 1,830 |
| #29 | telehealth.ti,ab. | Title/abstract | 15,186 |
| #30 | teleintervention.ti,ab. | Title/abstract | 23 |
| #31 | telemedicine.ti,ab. | Title/abstract | 22,766 |
| #32 | telemonitoring.ti,ab. | Title/abstract | 2,613 |
| #33 | telepsych*.ti,ab. | Title/abstract | 1,204 |
| #34 | telerehabilitation.ti,ab. | Title/abstract | 2,410 |
| #35 | telecommunication.ti,ab. | Title/abstract | 3,480 |
| #36 | ((telephone or phone) adj2 (app* or intervention* or therap* or system* or service* or support or tool* or program*)).ti,ab. | Title/abstract | 9,572 |
| #37 | (technolog* adj2 (intervention* or therap* or support or tool* or program*)).ti,ab. | Title/abstract | 17,511 |
| #38 | website*.ti,ab. | Title/abstract | 47,719 |
| #39 | (web adj2 (intervention* or program* or tool*)).ti,ab. | Title/abstract | 8,966 |
| #40 | or/8-39 | Combined | 386,962 |
| #41 | 3 and 7 and 40 | Combined | 388 |
| #42 | limit 41 to yr="1998 - 2025" | Limit | 386 |

**Embase (Elsevier) search strategy**

| **Line no** | **Search string** | **Search fields** | **Hits** |
| --- | --- | --- | --- |
| #1 | diabetes mellitus type 2'/exp | MeSh | 428,089 |
| #2 | ('T2DM' OR 'T2D' OR 'type 2 diabetes' OR 'type two diabetes' OR (non-insulin-dependent NEAR/2 diabetes) OR 'NIDDM' OR ('adult-onset' NEAR/2 diabetes)):ti,ab | Title/abstract | 330,401 |
| #3 | #1 OR #2 | Combined | 483,555 |
| #4 | 'psychological distress'/exp OR 'psychological well-being'/exp | MeSh | 124,238 |
| #5 | (diabetes NEAR/2 (distress OR stress OR self-care OR self-efficacy OR empowerment OR emotion* OR 'quality of life' OR well-being OR wellbeing OR anxiety OR depression)):ti,ab | Title/abstract | 14,501 |
| #6 | ((psychological OR psychosocial OR emotional OR psychophysical) NEAR/2 (distress OR stress OR burden OR well-being OR wellbeing OR health OR outcomes)):ti,ab | Title/abstract | 181,483 |
| #7 | 4 OR 5 OR 6 | Combined | 264,734 |
| #8 | telemedicine'/exp OR 'digital health'/exp OR 'virtual reality'/exp OR 'internet based intervention'/exp OR 'mobile application'/exp OR 'technology'/exp | MeSh | 464,163 |
| #9 | blog*:ti,ab | Title/abstract | 4,330 |
| #10 | (computer NEAR/2 (app* OR therap* OR intervention* OR program* OR communication*)):ti,ab | Title/abstract | 33,401 |
| #11 | cybertherapy:ti,ab | Title/abstract | 65 |
| #12 | (digital NEAR/2 (app* OR health OR intervention* OR service* OR solution* OR tool* OR program* OR technolog*)):ti,ab | Title/abstract | 38,632 |
| #13 | (eHealth OR e-Health):ti,ab | Title/abstract | 11,530 |
| #14 | e-mental health':ti,ab | Title/abstract | 566 |
| #15 | (e-counsel* OR ecounsel*):ti,ab | Title/abstract | 119 |
| #16 | (e-psychotherap* OR epsychotherap*):ti,ab | Title/abstract | 47 |
| #17 | email therapy':ti,ab | Title/abstract | 3 |
| #18 | forum*:ti,ab | Title/abstract | 30,923 |
| #19 | (internet NEAR/2 intervention*):ti,ab | Title/abstract | 2,601 |
| #20 | mHealth:ti,ab | Title/abstract | 9,448 |
| #21 | (mobile NEAR/2 (app* OR health OR support OR technolog* OR program* OR intervention* OR tool*)):ti,ab | Title/abstract | 36,564 |
| #22 | (online NEAR/2 (intervention* OR therap* OR system* OR service* OR support OR community OR tool* OR program*)):ti,ab | Title/abstract | 33,796 |
| #23 | (portable NEAR/2 (app* OR technolog*)):ti,ab | Title/abstract | 1,980 |
| #24 | (remote NEAR/2 (coach* OR counsel* OR psych* OR support OR intervention* OR monitor*)):ti,ab | Title/abstract | 15,370 |
| #25 | SMS:ti,ab | Title/abstract | 13,194 |
| #26 | messag*:ti,ab | Title/abstract | 124,447 |
| #27 | telecare:ti,ab | Title/abstract | 1,171 |
| #28 | teleconsultation:ti,ab | Title/abstract | 2,756 |
| #29 | telehealth:ti,ab | Title/abstract | 21,787 |
| #30 | teleintervention:ti,ab | Title/abstract | 65 |
| #31 | telemedicine:ti,ab | Title/abstract | 32,801 |
| #32 | telemonitoring:ti,ab | Title/abstract | 4,625 |
| #33 | telepsych*:ti,ab | Title/abstract | 1,675 |
| #34 | telerehabilitation:ti,ab | Title/abstract | 3,922 |
| #35 | telecommunication:ti,ab | Title/abstract | 3,692 |
| #36 | ((telephone OR phone) NEAR/2 (app* OR intervention* OR therap* OR system* OR service* OR support OR tool* OR program*)):ti,ab | Title/abstract | 16,365 |
| #37 | (technolog* NEAR/2 (intervention* OR therap* OR support OR tool* OR program*)):ti,ab | Title/abstract | 22,030 |
| #38 | website*:ti,ab | Title/abstract | 72,249 |
| #39 | (web NEAR/2 (intervention* OR program* OR tool*)):ti,ab | Title/abstract | 11,826 |
| #40 | #8 OR #9 OR #10 OR #11 OR #12 OR #13 OR #14 OR #15 OR #16 OR #17 OR #18 OR #19 OR #20 OR #21 OR #22 OR #23 OR #24 OR #25 OR #26 OR #27 OR #28 OR #29 OR #30 OR #31 OR #32 OR #33 OR #34 OR #35 OR #36 OR #37 OR #38 OR #39 | Combined | 842,278 |
| #41 | #3 AND #7 AND #40 | Combined | 826 |
| #42 | #41 AND [1998-2025]/py | Limit | 824 |

**CINAHL (EBSCO) search strategy**

| **Line no** | **Search string** | **Search fields** | **Hits** |
| --- | --- | --- | --- |
| #1 | (MH "Diabetes Mellitus, Type 2+") | MeSh | 73,779 |
| #2 | TI "T2DM" OR TI "T2D" OR TI "type 2 diabetes" OR TI "type two diabetes" OR TI "non-insulin-dependent diabetes" OR TI "NIDDM" OR TI "adult-onset diabetes" OR AB "T2DM" OR AB "T2D" OR AB "type 2 diabetes" OR AB "type two diabetes" OR AB "non-insulin-dependent diabetes" OR AB "NIDDM" OR AB "adult-onset diabetes" | Title/abstract | 67,694 |
| #3 | S1 OR S2 | Combined | 95,426 |
| #4 | (MH "Psychological Distress") OR (MH "Psychological Well-Being") | MeSh | 60,344 |
| #5 | TI diabetes N2 (distress OR stress OR self-care OR self-efficacy OR empowerment OR emotion* OR "quality of life" OR well-being OR wellbeing OR anxiety OR depression) OR AB diabetes N2 (distress OR stress OR self-care OR self-efficacy OR empowerment OR emotion* OR "quality of life" OR well-being OR wellbeing OR anxiety OR depression) | Title/abstract | 6,037 |
| #6 | TI (psychological OR psychosocial OR emotional OR psychophysical) N2 (distress OR stress OR burden OR well-being OR wellbeing OR health OR outcomes) OR AB (psychological OR psychosocial OR emotional OR psychophysical) N2 (distress OR stress OR burden OR well-being OR wellbeing OR health OR outcomes) | Title/abstract | 72,382 |
| #7 | S4 OR S5 OR S6 | Combined | 122,291 |
| #8 | (MH "Telemedicine") OR (MH "Digital Health") OR (MH "Virtual Reality") OR (MH "Internet-Based Intervention") OR (MH "Mobile Applications") OR (MH "Technology") | MeSh | 64,921 |
| #9 | TI blog* OR AB blog* | Title/abstract | 2,957 |
| #10 | TI (computer N2 (app* OR therap* OR intervention* OR program* OR communication*)) OR AB (computer N2 (app* OR therap* OR intervention* OR program* OR communication*)) | Title/abstract | 6,008 |
| #11 | TI (cybertherapy) OR AB (cybertherapy) | Title/abstract | 85 |
| #12 | TI (digital N2 (app* OR health OR intervention* OR service* OR solution* OR tool* OR program* OR technolog*)) OR AB (digital N2 (app* OR health OR intervention* OR service* OR solution* OR tool* OR program* OR technolog*)) | Title/abstract | 13,018 |
| #13 | TI (eHealth OR e-Health) OR AB (eHealth OR e-Health) | Title/abstract | 5,053 |
| #14 | TI (e-Mental Health) OR AB (e-Mental Health) | Title/abstract | 165 |
| #15 | TI (e-counsel* OR ecounsel*) OR AB (e-counsel* OR ecounsel*) | Title/abstract | 27 |
| #16 | TI (e-psychotherap* OR epsychotherap*) OR AB (e-psychotherap* OR epsychotherap*) | Title/abstract | 1 |
| #17 | TI ("email therapy") OR AB ("email therapy") | Title/abstract | 1 |
| #18 | TI (forum*) OR AB (forum*) | Title/abstract | 19,904 |
| #19 | TI (internet N2 intervention*) OR AB (internet N2 intervention*) | Title/abstract | 1,326 |
| #20 | TI (mHealth) OR AB (mHealth) | Title/abstract | 3,071 |
| #21 | TI (mobile N2 (app* OR health OR support OR technolog* OR program* OR intervention* OR tool*)) OR AB (mobile N2 (app* OR health OR support OR technolog* OR program* OR intervention* OR tool*)) | Title/abstract | 12,287 |
| #22 | TI (online N2 (intervention* OR therap* OR system* OR service* OR support OR community OR tool* OR program*)) OR AB (online N2 (intervention* OR therap* OR system* OR service* OR support OR community OR tool* OR program*)) | Title/abstract | 14,511 |
| #23 | TI (portable N2 (app* OR technolog*)) OR AB (portable N2 (app* OR technolog*)) | Title/abstract | 304 |
| #24 | TI (remote N2 (coach* OR counsel* OR psych* OR support OR intervention* OR monitor*)) OR AB (remote N2 (coach* OR counsel* OR psych* OR support OR intervention* OR monitor*)) | Title/abstract | 3,446 |
| #25 | TI (SMS) OR AB (SMS) | Title/abstract | 2,168 |
| #26 | TI (messag*) OR AB (messag*) | Title/abstract | 49,367 |
| #27 | TI (telecare) OR AB (telecare) | Title/abstract | 702 |
| #28 | TI (teleconsultation) OR AB (teleconsultation) | Title/abstract | 760 |
| #29 | TI (telehealth) OR AB (telehealth) | Title/abstract | 9,779 |
| #30 | TI (teleintervention) OR AB (teleintervention) | Title/abstract | 12 |
| #31 | TI (telemedicine) OR AB (telemedicine) | Title/abstract | 9,056 |
| #32 | TI (telemonitoring) OR AB (telemonitoring) | Title/abstract | 1,131 |
| #33 | TI (telepsych*) OR AB (telepsych*) | Title/abstract | 645 |
| #34 | TI (telerehabilitation) OR AB (telerehabilitation) | Title/abstract | 1,212 |
| #35 | TI (telecommunication) OR AB (telecommunication) | Title/abstract | 1,190 |
| #36 | TI ((telephone OR phone) N2 (app* OR intervention* OR therap* OR system* OR service* OR support OR tool* OR program*)) OR AB ((telephone OR phone) N2 (app* OR intervention* OR therap* OR system* OR service* OR support OR tool* OR program*)) | Title/abstract | 7,069 |
| #37 | TI (technolog* N2 (intervention* OR therap* OR support OR tool* OR program*)) OR AB (technolog* N2 (intervention* OR therap* OR support OR tool* OR program*)) | Title/abstract | 9,562 |
| #38 | TI (website*) OR AB (website*) | Title/abstract | 20,762 |
| #39 | TI (web N2 (intervention* OR program* OR tool*)) OR AB (web N2 (intervention* OR program* OR tool*)) | Title/abstract | 3,950 |
| #40 | S8 OR S9 OR S10 OR S11 OR S12 OR S13 OR S14 OR S15 OR S16 OR S17 OR S18 OR S19 OR S20 OR S21 OR S22 OR S23 OR S24 OR S25 OR S26 OR S27 OR S28 OR S29 OR S30 OR S31 OR S32 OR S33 OR S34 OR S35 OR S36 OR S37 OR S38 OR S39 | Combined | 217,254 |
| #41 | S3 AND S7 AND S40 | Combined | 232 |
| **#42** | **Limit S41 to 01/01/1998 - 07/08/2025** | **Limit** | **231** |

**PsycINFO (EBSCO) search strategy**

| **Line no** | **Search string** | **Search fields** | **Hits** |
| --- | --- | --- | --- |
| #1 | MA ("Diabetes Mellitus, Type 2") | MeSh | 5,122 |
| #2 | TI ("T2DM" OR "T2D" OR "type 2 diabetes" OR "type two diabetes" OR "non-insulin-dependent diabetes" OR "NIDDM" OR "adult-onset diabetes") OR AB ("T2DM" OR "T2D" OR "type 2 diabetes" OR "type two diabetes" OR "non-insulin-dependent diabetes" OR "NIDDM" OR "adult-onset diabetes") | Title/abstract | 9,742 |
| #3 | S1 OR S2 | Combined | 11,166 |
| #4 | (MA "Psychological Distress") OR (MA "Psychological Well-Being") | MeSh | 1,099 |
| #5 | TI diabetes N2 (distress OR stress OR self-care OR self-efficacy OR empowerment OR emotion* OR "quality of life" OR well-being OR wellbeing OR anxiety OR depression) OR AB diabetes N2 (distress OR stress OR self-care OR self-efficacy OR empowerment OR emotion* OR "quality of life" OR well-being OR wellbeing OR anxiety OR depression) | Title/abstract | 3,388 |
| #6 | TI (psychological OR psychosocial OR emotional OR psychophysical) N2 (distress OR stress OR burden OR well-being OR wellbeing OR health OR outcomes) OR AB (psychological OR psychosocial OR emotional OR psychophysical) N2 (distress OR stress OR burden OR well-being OR wellbeing OR health OR outcomes) | Title/abstract | 120,618 |
| #7 | S4 OR S5 OR S6 | Combined | 123,725 |
| #8 | (MA "Telemedicine") OR (MA "Digital Health") OR (MA "Virtual Reality") OR (MA "Internet-Based Intervention") OR (MA "Mobile Applications") OR (MA "Technology") | MeSh | 8,890 |
| #9 | TI blog* OR AB blog* | Title/abstract | 3,920 |
| #10 | TI (computer N2 (app* OR therap* OR intervention* OR program* OR communication*)) OR AB (computer N2 (app* OR therap* OR intervention* OR program* OR communication*)) | Title/abstract | 14,450 |
| #11 | TI (cybertherapy) OR AB (cybertherapy) | Title/abstract | 98 |
| #12 | TI (digital N2 (app* OR health OR intervention* OR service* OR solution* OR tool* OR program* OR technolog*)) OR AB (digital N2 (app* OR health OR intervention* OR service* OR solution* OR tool* OR program* OR technolog*)) | Title/abstract | 12,817 |
| #13 | TI (eHealth OR e-Health) OR AB (eHealth OR e-Health) | Title/abstract | 2,284 |
| #14 | TI (e-Mental Health) OR AB (e-Mental Health) | Title/abstract | 265 |
| #15 | TI (e-counsel* OR ecounsel*) OR AB (e-counsel* OR ecounsel*) | Title/abstract | 67 |
| #16 | TI (e-psychotherap* OR epsychotherap*) OR AB (e-psychotherap* OR epsychotherap*) | Title/abstract | 8 |
| #17 | TI ("email therapy") OR AB ("email therapy") | Title/abstract | 6 |
| #18 | TI (forum*) OR AB (forum*) | Title/abstract | 11,894 |
| #19 | TI (internet N2 intervention*) OR AB (internet N2 intervention*) | Title/abstract | 1,740 |
| #20 | TI (mHealth) OR AB (mHealth) | Title/abstract | 1,664 |
| #21 | TI (mobile N2 (app* OR health OR support OR technolog* OR program* OR intervention* OR tool*)) OR AB (mobile N2 (app* OR health OR support OR technolog* OR program* OR intervention* OR tool*)) | Title/abstract | 10,166 |
| #22 | TI (online N2 (intervention* OR therap* OR system* OR service* OR support OR community OR tool* OR program*)) OR AB (online N2 (intervention* OR therap* OR system* OR service* OR support OR community OR tool* OR program*)) | Title/abstract | 19,303 |
| #23 | TI (portable N2 (app* OR technolog*)) OR AB (portable N2 (app* OR technolog*)) | Title/abstract | 241 |
| #24 | TI (remote N2 (coach* OR counsel* OR psych* OR support OR intervention* OR monitor*)) OR AB (remote N2 (coach* OR counsel* OR psych* OR support OR intervention* OR monitor*)) | Title/abstract | 1,488 |
| #25 | TI (SMS) OR AB (SMS) | Title/abstract | 2,097 |
| #26 | TI (messag*) OR AB (messag*) | Title/abstract | 54,750 |
| #27 | TI (telecare) OR AB (telecare) | Title/abstract | 260 |
| #28 | TI (teleconsultation) OR AB (teleconsultation) | Title/abstract | 331 |
| #29 | TI (telehealth) OR AB (telehealth) | Title/abstract | 4,664 |
| #30 | TI (teleintervention) OR AB (teleintervention) | Title/abstract | 12 |
| #31 | TI (telemedicine) OR AB (telemedicine) | Title/abstract | 2,975 |
| #32 | TI (telemonitoring) OR AB (telemonitoring) | Title/abstract | 257 |
| #33 | TI (telepsych*) OR AB (telepsych*) | Title/abstract | 1,124 |
| #34 | TI (telerehabilitation) OR AB (telerehabilitation) | Title/abstract | 385 |
| #35 | TI (telecommunication) OR AB (telecommunication) | Title/abstract | 2,426 |
| #36 | TI ((telephone OR phone) N2 (app* OR intervention* OR therap* OR system* OR service* OR support OR tool* OR program*)) OR AB ((telephone OR phone) N2 (app* OR intervention* OR therap* OR system* OR service* OR support OR tool* OR program*)) | Title/abstract | 5,293 |
| #37 | TI (technolog* N2 (intervention* OR therap* OR support OR tool* OR program*)) OR AB (technolog* N2 (intervention* OR therap* OR support OR tool* OR program*)) | Title/abstract | 10,940 |
| #38 | TI (website*) OR AB (website*) | Title/abstract | 17,397 |
| #39 | TI (web N2 (intervention* OR program* OR tool*)) OR AB (web N2 (intervention* OR program* OR tool*)) | Title/abstract | 3,407 |
| #40 | S8 OR S9 OR S10 OR S11 OR S12 OR S13 OR S14 OR S15 OR S16 OR S17 OR S18 OR S19 OR S20 OR S21 OR S22 OR S23 OR S24 OR S25 OR S26 OR S27 OR S28 OR S29 OR S30 OR S31 OR S32 OR S33 OR S34 OR S35 OR S36 OR S37 OR S38 OR S39 | Combined | 166,263 |
| #41 | S3 AND S7 AND S40 | Combined | 110 |
| **#42** | **Limit S41 to 01/01/1998 - 07/08/2025** | **Limit** | **110** |

**Cochrane Library search strategy**

| **Line no** | **Search string** | **Search fields** | **Hits** |
| --- | --- | --- | --- |
| #1 | MeSH descriptor: [Diabetes Mellitus, Type 2] explode all trees | MeSh | 26,908 |
| #2 | ("T2DM" OR "T2D" OR "type 2 diabetes" OR "type two diabetes" OR (non-insulin-dependent NEAR/2 diabetes) OR "NIDDM" OR ("adult-onset" NEAR/2 diabetes)):ti,ab | Title/abstract | 50,315 |
| #3 | #1 OR #2 | Combined | 55,510 |
| #4 | MeSH descriptor: [Psychological Distress] explode all trees | MeSh | 640 |
| #5 | MeSH descriptor: [Psychological Well-being] explode all trees | MeSh | 78 |
| #6 | (diabetes NEAR/2 (distress OR stress OR self-care OR self-efficacy OR empowerment OR emotion* OR "quality of life" OR well-being OR wellbeing OR anxiety OR depression)):ti,ab | Title/abstract | 2,801 |
| #7 | ((psychological OR psychosocial OR emotional OR psychophysical) NEAR/2 (distress OR stress OR burden OR well-being OR wellbeing OR health OR outcomes)):ti,ab | Title/abstract | 22,727 |
| #8 | #4 OR #5 OR #6# OR #7 | Combined | 25,377 |
| #9 | MeSH descriptor: [Telemedicine] explode all trees | MeSh | 5,556 |
| #10 | MeSH descriptor: [Digital Health] explode all trees | MeSh | 67 |
| #11 | MeSH descriptor: [Virtual Reality] explode all trees | MeSh | 1,515 |
| #12 | MeSH descriptor: [Internet-Based Intervention] explode all trees | MeSh | 962 |
| #13 | MeSH descriptor: [Mobile Applications] explode all trees | MeSh | 2,523 |
| #14 | MeSH descriptor: [Technology] explode all trees | MeSh | 9,395 |
| #15 | blog*:ti,ab | Title/abstract | 192 |
| #16 | (computer NEAR/2 (app* OR therap* OR intervention* OR program* OR communication*)):ti,ab | Title/abstract | 3,427 |
| #17 | cybertherapy:ti,ab | Title/abstract | 4 |
| #18 | (digital NEAR/2 (app* OR health OR intervention* OR service* OR solution* OR tool* OR program* OR technolog*)):ti,ab | Title/abstract | 5,188 |
| #19 | (eHealth OR e-Health):ti,ab | Title/abstract | 2,108 |
| #20 | e-Mental Health:ti,ab | Title/abstract | 161 |
| #21 | (e-counsel* OR ecounsel*):ti,ab | Title/abstract | 47 |
| #22 | (e-psychotherap* OR epsychotherap*):ti,ab | Title/abstract | 10 |
| #23 | email therapy:ti,ab | Title/abstract | 6 |
| #24 | forum*:ti,ab | Title/abstract | 1,187 |
| #25 | (internet NEAR/2 intervention*):ti,ab | Title/abstract | 1,704 |
| #26 | mHealth:ti,ab | Title/abstract | 2,977 |
| #27 | (mobile NEAR/2 (app* OR health OR support OR technolog* OR program* OR intervention* OR tool*)):ti,ab | Title/abstract | 9,460 |
| #28 | (online NEAR/2 (intervention* OR therap* OR system* OR service* OR support OR community OR tool* OR program*)):ti,ab | Title/abstract | 6,698 |
| #29 | (portable NEAR/2 (app* OR technolog*)):ti,ab | Title/abstract | 93 |
| #30 | (remote NEAR/2 (coach* OR counsel* OR psych* OR support OR intervention* OR monitor*)):ti,ab | Title/abstract | 2,540 |
| #31 | SMS:ti,ab | Title/abstract | 3,317 |
| #32 | messag*:ti,ab | Title/abstract | 18,050 |
| #33 | telecare:ti,ab | Title/abstract | 266 |
| #34 | teleconsultation:ti,ab | Title/abstract | 234 |
| #35 | telehealth:ti,ab | Title/abstract | 3,358 |
| #36 | teleintervention:ti,ab | Title/abstract | 37 |
| #37 | telemedicine:ti,ab | Title/abstract | 2,883 |
| #38 | telemonitoring:ti,ab | Title/abstract | 1,314 |
| #39 | telepsych*:ti,ab | Title/abstract | 165 |
| #40 | telerehabilitation:ti,ab | Title/abstract | 1,717 |
| #41 | telecommunication:ti,ab | Title/abstract | 196 |
| #42 | ((telephone OR phone) NEAR/2 (app* OR intervention* OR therap* OR system* OR service* OR support OR tool* OR program*)):ti,ab | Title/abstract | 6,328 |
| #43 | (technolog* NEAR/2 (intervention* OR therap* OR support OR tool* OR program*)):ti,ab | Title/abstract | 2,884 |
| #44 | website*:ti,ab | Title/abstract | 7,581 |
| #45 | (web NEAR/2 (intervention* OR program* OR tool*)):ti,ab | Title/abstract | 2,551 |
| #46 | #9 OR #10 OR #11 OR #12 OR #13 OR #14 OR #15 OR #16 OR #17 OR #18 OR #19 OR #20 OR #21 OR #22 OR #23 OR #24 OR #25 OR #26 OR #27 OR #28 OR #29 OR #30 OR #31 OR #32 OR #33 OR #34 OR #35 OR #36 OR #37 OR #38 OR #39 OR #40 OR #41 OR #42 OR #43 OR #44 OR #45 | Combined | 77,555 |
| #47 | #3 AND #8 AND #46 | Combined | 435 |
| **#48** | **Cochrane Library publication date from Jan 1998 to Aug 2025** | **Limit** | **435** |

**Epistemonikos search strategy**

| **Line no** | **Search string** | **Search fields** | **Hits** |
| --- | --- | --- | --- |
| #1 | (title:(("type 2 diabetes" OR T2DM OR T2D OR "type two diabetes" OR "non-insulin-dependent diabetes" OR NIDDM OR "adult-onset diabetes")) OR abstract:(("type 2 diabetes" OR T2DM OR T2D OR "type two diabetes" OR "non-insulin-dependent diabetes" OR NIDDM OR "adult-onset diabetes"))) | Title/abstract | 79,958 |
| #2 | (title:("psychological distress" OR "psychological well-being" OR "diabetes distress" OR "diabetes stress" OR "diabetes self-care" OR "diabetes self-efficacy" OR "diabetes empowerment" OR emotion* OR "quality of life" OR well-being OR wellbeing OR anxiety OR depression OR "psychological burden" OR "psychosocial burden" OR "emotional burden" OR "emotional stress" OR "psychosocial stress" OR "emotional health" OR "psychological outcomes" OR "emotional outcomes" OR "psychosocial outcomes" OR "psychophysical distress") OR abstract:("psychological distress" OR "psychological well-being" OR "diabetes distress" OR "diabetes stress" OR "diabetes self-care" OR "diabetes self-efficacy" OR "diabetes empowerment" OR emotion* OR "quality of life" OR well-being OR wellbeing OR anxiety OR depression OR "psychological burden" OR "psychosocial burden" OR "emotional burden" OR "emotional stress" OR "psychosocial stress" OR "emotional health" OR "psychological outcomes" OR "emotional outcomes" OR "psychosocial outcomes" OR "psychophysical distress")) | Title/abstract | 519,820 |
| #3 | (title:("digital health" OR "virtual reality" OR "internet-based intervention" OR "mobile applications" OR technology OR blog* OR (computer AND (app* OR therap* OR intervention* OR program* OR communication*)) OR cybertherapy OR (digital AND (app* OR health OR intervention* OR service* OR solution* OR tool* OR program* OR technolog*)) OR eHealth OR "e-Health" OR "e-Mental Health" OR eMentalHealth OR ecounsel* OR "e-counsel*" OR epsychotherap* OR "e-psychotherap*" OR "email therapy" OR forum* OR (internet AND intervention*) OR mHealth OR (mobile AND (app* OR health OR support OR technolog* OR program* OR intervention* OR tool*)) OR (online AND (intervention* OR therap* OR system* OR service* OR support OR community OR tool* OR program*)) OR (portable AND (app* OR technolog*)) OR (remote AND (coach* OR counsel* OR psych* OR support OR intervention* OR monitor*)) OR SMS OR messag* OR telecare OR teleconsultation OR telehealth OR teleintervention OR telemedicine OR telemonitoring OR telepsych* OR telerehabilitation OR telecommunication OR (telephone AND (app* OR intervention* OR therap* OR system* OR service* OR support OR tool* OR program*)) OR (phone AND (app* OR intervention* OR therap* OR system* OR service* OR support OR tool* OR program*)) OR (technolog* AND (intervention* OR therap* OR support OR tool* OR program*)) OR website* OR (web AND (intervention* OR program* OR tool*))) OR abstract:("digital health" OR "virtual reality" OR "internet-based intervention" OR "mobile applications" OR technology OR blog* OR (computer AND (app* OR therap* OR intervention* OR program* OR communication*)) OR cybertherapy OR (digital AND (app* OR health OR intervention* OR service* OR solution* OR tool* OR program* OR technolog*)) OR eHealth OR "e-Health" OR "e-Mental Health" OR eMentalHealth OR ecounsel* OR "e-counsel*" OR epsychotherap* OR "e-psychotherap*" OR "email therapy" OR forum* OR (internet AND intervention*) OR mHealth OR (mobile AND (app* OR health OR support OR technolog* OR program* OR intervention* OR tool*)) OR (online AND (intervention* OR therap* OR system* OR service* OR support OR community OR tool* OR program*)) OR (portable AND (app* OR technolog*)) OR (remote AND (coach* OR counsel* OR psych* OR support OR intervention* OR monitor*)) OR SMS OR messag* OR telecare OR teleconsultation OR telehealth OR teleintervention OR telemedicine OR telemonitoring OR telepsych* OR telerehabilitation OR telecommunication OR (telephone AND (app* OR intervention* OR therap* OR system* OR service* OR support OR tool* OR program*)) OR (phone AND (app* OR intervention* OR therap* OR system* OR service* OR support OR tool* OR program*)) OR (technolog* AND (intervention* OR therap* OR support OR tool* OR program*)) OR website* OR (web AND (intervention* OR program* OR tool*)))) | Title/abstract | 404,045 |
| #4 | #1 AND #2 AND #3 | Combination | 1237 |
| **#5** | **[Filters: protocol=no, min_year=1998, max_year=2025]** | **Limit** | **1236** |

**Europe PMC search strategy**

| **Line no** | **Search string** | **Search fields** | **Hits** |
| --- | --- | --- | --- |
| #1 | TITLE_ABS:(("type 2 diabetes" OR T2DM OR T2D OR NIDDM)) | Title/abstract | 217,239 |
| #2 | TITLE_ABS:("diabetes distress" OR "psychological distress" OR "depression" OR "anxiety" OR "quality of life") | Title/abstract | 2,725,787 |
| #3 | TITLE_ABS:(("digital health" OR eHealth OR telemedicine OR "internet-based intervention" OR "mobile application" OR mHealth OR online OR web OR telehealth)) | Title/abstract | 633,581 |
| #4 | #1 AND #2 # AND #3 | Combined | 1,849 |
| **#5** | **FIRST_PDATE:[1998-01-01 TO 2025-08-08]** | **Limit** | **1,849** |

**Trip PRO search strategy**

| **Line no** | **Search string** | **Search fields** | **Hits** |
| --- | --- | --- | --- |
| #1 | (title:"type 2 diabetes ") | Title | 50950 |
| #2 | (title:"adult onset diabetes") | Title | 42 |
| #3 | (title:"t2dm") | Title | 988 |
| #4 | (title:"t2d") | Title | 268 |
| #5 | (title:"non insulin dependent diabetes ") | Title | 615 |
| #6 | (title:"niddm ") | Title | 50950 |
| #7 | (title:"type two diabetes ") | Title | 53 |
| #8 | #1 OR #2 OR #3 OR #4 OR #5 OR #6 OR #7 OR #8 | Combined | 52219 |
| #9 | (title:"diabetes distress") | Title | 237 |
| #10 | (title:"psychological distress ") | Title | 2935 |
| #11 | ((title:depression)) | Title | 75929 |
| #12 | ((title:anxiety)) | Title | 30593 |
| #13 | (title:"quality of life") | Title | 42885 |
| #14 | ((title:wellbeing OR title:well-being)) | Title | 11710 |
| #15 | #9 OR #10 OR #11 OR #12 OR #13 OR #14 | Combined | 150827 |
| #16 | (title:"digital health") | Title | 1969 |
| #17 | (title:"ehealth") | Title | 1553 |
| #18 | (title:"telemedicine ") | Title | 3608 |
| #19 | (title:"internet-based intervention") | Title | 295 |
| #20 | (title:"mobile application") | Title | 1244 |
| #21 | (title:"mhealth") | Title | 2221 |
| #22 | (title:"online") | Title | 11682 |
| #23 | (title:"web") | Title | 8763 |
| #24 | (title:"technology") | Title | 24748 |
| #25 | (title:"telehealth") | Title | 3044 |
| #26 | #16 OR #17 OR #18 OR #19 OR #20 OR #21 OR #22 OR #23 OR #24 OR #25 | Combined | 57784 |
| #27 | #8 AND #15 AND #26 | Combined | 25 |
| #28 | from_date:1998 to_date:2025 | Limit | **25** |

**OTseeker search strategy**

| **Line no** | **Search string** | **Search fields** | **Hits** |
| --- | --- | --- | --- |
| #1 | [Any Field] like '("type 2 diabetes" OR t2dm OR t2d OR "non insulin dependent diabetes" OR niddm OR "adult onset diabetes")' | Any field | 145 |
| #2 | [Any Field] like '"diabetes distress" OR "psychological distress" OR depression OR anxiety OR "quality of life" OR wellbeing OR "well-being"' | Any field | 2639 |
| #3 | [Any Field] like '("digital health" OR ehealth OR telemedicine OR "internet-based intervention" OR "mobile application" OR mhealth OR online OR web OR technology OR telehealth)' | Any field | 699 |
| #4 | [Any Field] like '("type 2 diabetes" OR t2dm OR t2d OR "non insulin dependent diabetes" OR niddm OR "adult onset diabetes")' AND [Any Field] like '("diabetes distress" OR "psychological distress" OR depression OR anxiety OR "quality of life" OR wellbeing OR "well-being")' AND [Any Field] like '("digital health" OR ehealth OR telemedicine OR "internet-based intervention" OR "mobile application" OR mhealth OR online OR web OR technology OR telehealth)' | Combined | **2** |
